# Real-World Selection of Patients for Allogeneic HCT at a Single Centre: Lack of a Suitable Donor and Other Reasons for Not Proceeding

**DOI:** 10.3390/curroncol32090483

**Published:** 2025-08-29

**Authors:** Madeline Monaghan, An Duong, Kalina Abrol, Trang Doan, Carolina Cieniak, Harold Atkins, Natasha Kekre, Ashish Masurekar, Ram Vasudevan Nampoothiri, Santhosh Thyagu, Christopher N. Bredeson, Michael Kennah, David S. Allan

**Affiliations:** 1 Faculty of Medicine, University of Ottawa, Ottawa, ON K1H 8M5, Canadasthyagu@toh.ca (S.T.);; 2 Cancer Research Program, Ottawa Hospital Research Institute, Ottawa, ON K1H 8L6, Canada; 3 Transplantation & Cellular Therapy Program, Department of Medicine (Hematology), The Ottawa Hospital, Ottawa, ON K1H 8L6, Canada

**Keywords:** hematopoietic cell transplant, allogeneic, donor, no donor, search, co-morbidity, toxicity, disease progression

## Abstract

The reasons why patients cannot proceed with HCT, including cases where no suitable donor is identified, remain poorly described. We reviewed all referrals for allogeneic HCT to our programme between 1 January 2019 and 31 December 2023. Of 880 patients referred for allogeneic HCT, 494 (61.8%) proceeded to transplant (mean 52 ± 14.8 years, 61.5% male) using HLA-matched unrelated (64.2%) or related (19.4%) donors and HLA-mismatched (13%) or haploidentical (3%) donors. Of patients that did not proceed with HCT (386, 38.2%), disease-related causes (54.2%), patient preference (15.8%), and significant patient comorbidity (11.4%) were the most common reasons. Eleven patients (2.9% of transplants that did not proceed; 1.3% of all referrals) lacked a suitable donor and had HLA phenotypes most associated with Caucasian (six patients, 55%), First Nations, Inuit or Metis (two patients, 18%), Black African, Caribbean or African American (one patient, 9%), Asian or Pacific Islander (9%), or unknown ethnicity (one patient, 9%). Very few patients were unable to proceed with transplant due to lack of a suitable donor; however, those cases are overrepresented by non-Caucasian ethnicity relative to the population.

## Simple Summary

The reasons why patients cannot proceed with blood stem cell transplantation can include cases where no suitable donor is identified, but exactly how often this occurs can be hard to determine. By reviewing all patients referred for allogeneic transplant at our centre between 1 January 2019 and 31 December 2023, we can capture all patients that did not proceed to transplant for various reasons, including the lack of a donor. Of 880 patients referred for allogeneic transplant, 386 (38.2%) did not proceed with transplant, but only a small number (11, or 2.9%, of all those who did not proceed and just 1.3% of all referrals) lacked a suitable donor. These patients were more often non-Caucasian compared with those undergoing transplant. Overall, very few patients were unable to proceed with transplant due to the lack of a suitable donor; however, greater focus on broad ethnic diversity in recruitment to stem cell registries may increase donor options further for these “hard to match” patients.

## 1. Introduction

Allogeneic hematopoietic cell transplantation (HCT) is a potentially curative treatment for various hematologic malignancies and other life-threatening disorders [1,2]. Successful transplantation depends on the identification of a suitable donor, which can be a human leukocyte antigen (HLA)-matched sibling, a matched or mismatched unrelated donor, a related haploidentical (half-matched) donor, or an umbilical cord blood unit [3]. With an increasing global inventory of unrelated donor registrants and improved transplant strategies that can allow improved outcomes with mismatched and haploidentical donors, more patients are eligible to proceed with HCT [4,5]. Despite these advancements, some patients referred for allogeneic HCT are unable to proceed with transplant due to lack of a suitable donor and other reasons.

The lack of a suitable donor remains a significant concern, particularly for patients from racial and ethnic minority backgrounds, who are less likely to find a fully matched unrelated donor [6]. Previous studies have shown that individuals of European descent have a significantly higher probability of finding a HLA-matched donor compared with those of African, Asian, Hispanic, or Native American ancestry. This reduced HLA-match likelihood arises because of lower-frequency HLA phenotypes among several non-Caucasian ethnic groups [7] and is compounded by reduced proportional representation within global donor registries [8]. Our goal was to understand the extent to which a lack of a suitable donor contributed to the inability of patients to proceed with HCT. Understanding the factors associated with a lack of a suitable donor can help inform targeted donor recruitment strategies and improve transplant accessibility. Our report offers novel insight on a general transplant population in a publicly funded health care system offering universal access to transplant and in a country with broad ethnic diversity.

## 2. Methods

### 2.1. Study Population and Design

This was a single centre, retrospective cohort study. All referrals for a first transplant to the allogeneic HCT programme at the Ottawa Hospital in Ontario, Canada, between 1 January 2019 and 31 December 2023, were included for analysis. Patients referred for consideration of a second allogeneic transplant were excluded.

### 2.2. Data Analysis

Descriptive statistics, including mean and standard deviation, were used to describe transplant-related outcomes. Microsoft Excel (Microsoft Corp, Seattle, DC, USA) was used for all data analyses, including the calculation of *t*-tests, chi-square tests, and *p*-values.

### 2.3. Outcomes

We retrieved basic demographic data including age at referral, sex, dates of referral for consultation for allogeneic HCT, date patient was removed from the care list or proceeded to transplant, and disease indication for transplant. As needed, patients were reassessed by the referring centres and/or our transplant team and discussed during planned weekly meetings with the referring centres and through additional communications prior to determining a final plan, which in some cases resulted in not proceeding to transplant. For patients that proceeded to transplant, we retrieved the donor type used for transplant (HLA-matched sibling donor, HLA-matched unrelated donor, HLA-mismatched unrelated donor, or haploidentical donor) if they proceeded with HCT. We determined the number of patients in each category from a list of six cancellation reasons: (1) disease-related, (2) patient comorbidity that precluded proceeding with transplant, (3) the lack of a suitable donor, (4) treatment-related toxicity that precluded proceeding with transplant, (5) patient preference (includes patients who were uncomfortable with the associated risks of transplant and opted against it, despite the willingness of the transplant team to proceed with transplant), (6) patient deceased after consultation but prior to performing a planned transplant, or a combination of these reasons (categories formalized by Ontario Health, Stem Cell committee).

We performed a planned subgroup analysis for the group where transplant was canceled due to the lack of a suitable donor. A chart review was performed to determine patients’ HLA type, the presence of siblings, and whether the siblings were HLA typed. We used patients’ HLA phenotypes to determine the most likely associated ethnicities from the public dataset from the National Marrow Donor Program (www.haplostats.org (see below)).

### 2.4. Haplostats

In the group that was canceled due to the lack of a suitable donor, we accessed a dataset of HLA phenotypes from the National Marrow Donor Program (www.haplostats.org; accessed on 15 January 2025) to determine the ethnicity groups that were most likely associated with their specific HLA phenotypes. Frequencies of specific HLA phenotypes are reported for the following ethnicities: Caucasian (includes Middle Eastern or North Coast of Africa and European Caucasian), Native American (includes North American Indian), African American (includes African American, African, and Caribbean Black), Asian or Pacific Islander (includes South Asian Indian, Filipino, Hawaiian or other Pacific Islander, Japanese, Korean, Chinese, Southeast Asian, and Vietnamese). For each patient, we listed only the ethnicity with the highest HLA frequency (i.e., most likely ethnicity).

### 2.5. Duplicates

Some patients were referred for transplant more than once during the study period. When there was more than one referral, we used the most recent referral only.

### 2.6. Donor Search Algorithm

Our donor selection algorithm follows the following general approach but can be modified on a case-by-case basis. Siblings that are willing, generally available, well, pass a screening eligibility assessment, and are ≤65 years of age are considered and invited by the recipient to contact us and undergo HLA typing. In some cases, siblings > 65 years of age are also considered. A search of unrelated donors is initiated in parallel in cases considered urgent (i.e., acute leukemia) but can be initiated following the results of sibling typing in other cases. While a HLA-matched donor is preferred (related or unrelated), a mismatched unrelated donor may be considered as an alternative option for most patients. Occasionally, mismatched unrelated donors are not pursued if additional patient or disease factors introduce a level of risk that is deemed excessive. When multiple matched donor options exist, we prioritize young donor age (≤30 years) and other factors, including CMV serostatus, ABO compatibility, weight discrepancy, and availability and scheduling. Haploidentical donors are considered if reduced-intensity conditioning is appropriate. Our centre does not perform umbilical cord blood transplantation but will consider cord blood on rare case-by-case situations and can refer to other regional centres with adult umbilical cord blood transplantation experience. We have developed common procedures for all our common referral centres that includes HLA typing in all cases of acute leukemia at the time of diagnosis. In other cases, HLA typing is performed when allogeneic HCT is considered as an option. Unrelated donor searches remain open and are updated weekly throughout the period when allogeneic HCT is being considered by our national donor registry. We do not have a specific period when donor searches are canceled, but instead, we remove patients from the active care list and cancel donor searches when a definitive plan has been finalized, which can include not proceeding with transplant due to any of the reasons listed in Methods, including the lack of a suitable donor. The criteria for donor suitability, or course, can change from one patient to another. When the most favourable donor option is combined with other patient and disease factors, our group, together with patients, arrives at the final decision to not proceed with transplant. In some cases, we may not proceed with a mismatched unrelated donor for a specific patient, whereas we may proceed with a similarly mismatched donor for a different patient. The availability of a HLA-matched donor would preclude classification as “lack of a suitable donor”.

## 3. Results

### 3.1. Characteristics of the Study Population

A total of 872 patients were referred to the allogeneic HCT programme between 1 January 2019 and 31 December 2023. Of the 872 referrals, 56.7% proceeded to transplant (n = 494), and 43.3% did not proceed to transplant (n = 378) and were listed as “canceled” (Table 1).

The mean age at referral for all patients was 54 ± 15 years (61.9% male). The mean age of patients undergoing transplant was 52 ± 14.8 (61.5% male), while the mean age of the group that did not proceed with transplant was 58 ± 13.0 (62.1% male) (*p* < 0.001) (Table 1).

The most frequent indication for transplant was acute myeloid leukemia (AML). Of the total referrals, 40.3% were for AML (n = 351). Other common indications were myelodysplastic syndrome (MDS) (22.9%, n = 200) and acute lymphoblastic leukemia (ALL) (13.4%, n = 117). All other disease indications for transplant are listed in Table 1.

### 3.2. Transplanted Patients

The most common indication for transplant in the study group was AML (43.32%, n = 214), followed by MDS (24.49%, n = 121) and ALL (15.79%, n = 78) (Table 1). Other indications are included in Table 1. The types of donors used at our centre included HLA-matched unrelated (64.2%), HLA-matched related (19.4%), HLA-mismatched unrelated (13%), and haploidentical donors (3.0%) with one HLA-mismatched related donor (0.40%) (Table 2).

The mean time interval between referral and consult was 33.9 ± 49 days in all patients who proceeded to transplant. The mean time interval between consult and transplant was 122 ± 93 days (Table 1). In patients with AML or ALL who underwent transplant, the time between referral and consult and between consult to transplant was not different.

### 3.3. Referrals That Did Not Proceed to Transplant

Of the 872 patients referred to the allogeneic HCT programme in the study period, 378 (43.2%) patients did not proceed to transplant. The reasons for not proceeding are listed in Table 3. The most common reason patients did not proceed to transplant was disease-related in 203 patients (53.7% of all canceled transplants). The next most common reasons were patient preference (n = 61, 16.1%), patient comorbidity (n = 44, 11.6%), and patient deceased (n = 37, 9.8%). There was a total of 11 patients (2.4%) who did not proceed to transplant due to the lack of a suitable donor. In the group of 203 patients who did not proceed to transplant for disease-related reasons, the most common diseases were AML (n = 70, 34.5%), MDS (n = 39, 19.2%), and ALL (n = 27, 13.3%).

In the subgroup analysis of the 11 patients who did not proceed to transplant due to donor unavailability, the mean age in this group was 66.5 ± 9.2 (*p* = 0.001 in comparison with age of transplanted group) (64% male) (Table 4). Six of these patients had AML (one patient with AML and acute myelofibrosis), two had MDS, and three had CML (one patient with concomitant MDS). When charts were reviewed for these 11 patients, 10 (90.9%) had living siblings, and 4 (36.4%) had siblings who underwent HLA typing (Table 4). It is not clear whether the siblings were assessed for eligibility.

The most associated ethnicities in the group who did not proceed to transplant due to the lack of a suitable donor were Caucasian (n = 6, 54.6%), Native American (2, 18.2%), African American (1, 9.1%), Asian or pacific islander (1, 9.1%), and one patient (9.1%) who had a HLA type that was not found in the Haplostats dataset (Table 4).

## 4. Discussion

Our study provides insights on reasons why patients do not proceed to transplant and concludes that the lack of a suitable donor is uncommon. The most common reason for not proceeding was disease-related factors, followed by patient preference and comorbidities that precluded proceeding to transplant. Notably, only 2.9% of canceled transplants were attributed to the inability to find a suitable donor, suggesting that donor availability is not a major limiting factor in our patient population. These findings are consistent with previous studies demonstrating that most patients referred for allogeneic HCT can find a suitable donor, particularly with the increasing utilization of haploidentical and mismatched unrelated donors [9], which accounted for an important number of patients in our study. By reporting on all patients referred for HCT and systematically reporting on the reasons for not proceeding to transplant, our report offers a robust and timely real-world experience in a publicly funded health care environment with broad ethnic diversity.

Our results, however, highlight a small subgroup of patients for whom donor availability remains a barrier. The demographic analysis of this subgroup suggests that while donor unavailability affects individuals from diverse ethnic backgrounds, patients of Native American, African American, and Asian/Pacific Islander descent may face greater challenges in securing a suitable donor. Non-Caucasian patients comprised 45% of the group that could not identify a suitable donor yet make up just 30.2% of the population in the 2021 census [10] and just 23.9% of patients in Canada looking for donors and where ethnicity was known [11]. This finding aligns with prior research indicating that ethnic minority populations are underrepresented in global donor registries, leading to disparities in donor availability [8]. Studies have shown that individuals of European descent have a higher likelihood of finding a fully matched unrelated donor compared with those from non-European backgrounds [12]. This discrepancy is largely due to differences in HLA allele frequencies across populations, emphasizing the need for targeted donor recruitment efforts. This group of patients that did not proceed due to the lack of a suitable donor were older, and while most had siblings, only a minority underwent HLA typing, suggesting that siblings of older patients may be more likely to be ineligible as donors. An additional study with more patients is needed.

Although our findings suggest that donor unavailability is a relatively infrequent reason that patients cannot proceed with transplant, continued efforts to enhance donor recruitment remain crucial, with the goal of finding HLA-matched options for more patients. While we did not characterize the most likely associated ethnicity of patients undergoing HCT with HLA-mismatched or haploidentical donors, a significantly higher proportion of non-Caucasian Canadian patients undergoing unrelated donor HCT required a MMUD (37%) or cord blood unit (9%) in a recent report [13] compared with Caucasian patients (14% and 2%, respectively), suggesting that access to optimal donor options remains a challenge for patients of non-Caucasian ethnicity. Information on haploidentical transplants is less clear. Strategies such as community outreach, educational initiatives, and the expansion of haploidentical donor programmes may help address disparities in donor availability [14]. Recent studies have demonstrated the effectiveness of such interventions in increasing donor pool diversity and improving transplant accessibility for underrepresented populations [6,15,16]. While targeted recruitment of specific ethnicities to registries may increase HLA-match likelihoods for some patients, the overall match likelihoods for all patients may not increase [17].

With regard to other reasons that patients cannot proceed to transplant, it is well recognized that patients must be “fit” for transplant and willing to embrace short-term risks that can impact mortality and quality of life. Even with reduced-intensity conditioning regimens that may allow older patients and those with comorbidities to undergo transplant, it remains a challenge for some patients, and transplant-related mortality can exceed 20% in patients with poor performance statuses and high HCT-CI scores that provide an objective measure of comorbidity burden and in frail patients [18]. And to prevent exposing patients to excessive rates of relapse following HCT, patients with acute leukemia with active disease are often excluded from proceeding, although new approaches may help to provide options for these patients [19].

Our study has several limitations. First, the use of public datasets of HLA phenotypes to infer patient ethnicity is an indirect method that may not accurately capture self-identified ethnicity. This limitation has been acknowledged in previous research, where HLA-based ancestry estimation has shown varying degrees of accuracy depending on population structure [7]. Additionally, our analysis is limited to a single transplant centre, which may not reflect trends observed at other institutions or within different geographic regions. Our study was underpowered to compare the clinical outcomes of patients who underwent transplant with patients in the groups who did not proceed to transplant. Our definition of a “suitable donor” infers a subjective element of shared decision-making between our team and the patient when an HLA-matched donor is not available, and we admit that this may differ between patients and from centre to centre. Moreover, the search for donors was extensive but not exhaustive and, generally, did not include a search for cord blood units. Future studies incorporating multi-center data and direct patient-reported ethnicity could provide a more comprehensive understanding of donor availability challenges. Also, a prospective study could continue to monitor patients and compare outcomes between those transplanted and those who did not proceed and even patients who declined a referral. In our real-world analysis, patients returned to their referring centres once removed from the transplant care list, and their clinical outcomes were not captured.

## 5. Conclusions

In conclusion, our findings suggest that most patients referred for allogeneic HCT successfully proceeded to transplant, with the lack of a suitable donor accounting for a small proportion of cases that did not proceed. While our current donor recruitment strategies appear effective, targeted efforts to increase donor diversity may further reduce barriers to transplant for underrepresented populations and increase the degree of HLA-matching and allow for the optimization of other non-HLA factors for all patients. Expanding donor registries and improving access to alternative donor sources, such as haploidentical, mismatched unrelated adult donors, and umbilical cord blood may help ensure that all patients have an equitable opportunity to receive life-saving transplants.

## Figures and Tables

**Table 1:** Summary of patients referred for allogeneic transplant between 1 January 2019 and 31 December 2023.

	All Patients	Transplant Group	Canceled Group	*p*-Value
Total patients, n	872	494	378	
Age at referral, mean yrs (SD)	54 (15.0)	52 (14.8)	58 (13.0)	<0.001
Sex (% male)	61.9	61.5	62.1	-
Time intervals in transplant process, mean days (SD)				
Referral to consult, mean days (SD) *	35.8 (46.4)	33.9 (49.0)	35.1 (45.8)	0.713
Consult to cancel, mean days (SD) *	-	-	90.5 (131.3)	-
Consult to transplant, mean days (SD)	-	122 (93.5)	-	-
Number of patients by disease, n (%)				
Acute myeloid leukemia	351 (40.3)	214 (43.3)	137 (36.2)	0.03
Myelodysplastic syndrome	200 (22.9)	121 (24.5)	79 (20.9)	0.21
Acute lymphoblastic leukemia	117 (13.4)	78 (15.8)	39 (10.3)	0.02
Non-Hodgkin lymphoma	47 (5.4)	19 (3.8)	28 (7.4)	0.02
Myeloproliferative neoplasms	41 (4.7)	15 (3.0)	26 (6.9)	0.01
Chronic myeloid leukemia	32 (3.7)	12 (2.4)	20 (5.3)	0.03
Severe aplastic anemia	25 (2.9)	13 (2.6)	12 (3.2)	0.63
Chronic lymphocytic leukemia	21 (2.4)	9 (1.8)	12 (3.2)	0.20
Hodgkin lymphoma	13 (1.5)	5 (1.0)	8 (2.1)	0.18
Sickle cell anemia	4 (0.5)	2 (0.4)	2 (0.5)	0.79
Multiple myeloma	4 (0.5)	0	4 (1.1)	0.02
Other	17 (2.0)	6 (1.2)	11 (2.9)	0.07

* A total of 38 patients were excluded in the analyses of “Time Between Referral & Consult” and “Time Between Consult & Cancellation” as they canceled before consult.

**Table 2:** Summary of donors for transplanted patients (n = 494).

Donor Type	Number of Patients (%)
Matched unrelated	317 (64.2%)
Matched related	96 (19.4%)
Mismatch unrelated	64 (13.0%)
9/10 match	63 (12.8%)
8/10 match	1 (0.2%)
Haploidentical (related)	15 (3.0%)
Mismatch related (both 9/10)	2 (0.4%)

**Table 3:** Summary of reasons why patients did not proceed to transplant (n = 378).

Indication for Cancellation	Number of Patients (%)
Disease-related	203 (53.7)
Patient preference	61 (16.1)
Patient comorbidity *	45 (11.9)
Patient deceased	37 (9.9)
Treatment toxicity **	13 (3.4)
Donor unavailability ***	11 (2.9)
Transfer of care/Treatment at another centre	6 (1.6)
Other reason (not reported)	2 (0.5)

* Includes 1 patient with comorbidity- and disease-related reasons for not proceeding. ** Includes 1 patient with treatment toxicity and comorbidity as reasons for not proceeding. *** Includes 2 patients classified as not proceeding due to the lack of suitable donor and patient preference.

**Table 4:** Summary of patients who did not proceed to transplant due to lack of a suitable donor (n = 11).

Patient Characteristic	%
Mean age, years (SD) (%)	66.5 (9.2)
Sex (% male)	64%
Number of patients by disease, n (%)	
Acute myeloid leukemia	4 (36%)
Myelodysplastic syndrome	3 (27%)
Chronic myeloid leukemia	2 (18%)
Myelofibrosis with acute leukemia	1 (9%)
Living sibling(s) (≥1 alive), n (%)	10 (91%)
Sibling(s) (≥1) HLA typed	4 (36%)
Ethnicity of patient *, n (%)	
Caucasian	6 (55%)
Native American	2 (18%)
African American	1 (9%)
Asian or Pacific Islander	1 (9%)
Loci not found/unable to assess	1 (9%)

* Data obtained from determining HLA frequencies through Haplostats. Ethnicity associations follow these groupings: Caucasian: includes Middle Eastern or North Coast of Africa and European Caucasian; Native American: includes North American Indian and Caribbean Indian and aligns closely with First Nations, Métis, and Inuit people within Canada; African American: includes African American, African, and Caribbean Black; Asian or Pacific Islander: includes South Asian Indian, Filipino, Hawaiian or other Pacific Islander, Japanese, Korean, Chinese, Southeast Asian, and Vietnamese.

## Data Availability

The datasets generated during and/or analyzed during the current study are available from the corresponding author on reasonable request.
